# Moisture barrier properties of thin organic-inorganic multilayers prepared by plasma-enhanced ALD and CVD in one reactor

**DOI:** 10.1186/1556-276X-9-223

**Published:** 2014-05-07

**Authors:** Tim Bülow, Hassan Gargouri, Mirko Siebert, Rolf Rudolph, Hans-Hermann Johannes, Wolfgang Kowalsky

**Affiliations:** 1 Technische Universität Braunschweig, Institut für Hochfrequenztechnik, Schleinitzstraße 22, Braunschweig 38106, Germany; 2 SENTECH Instruments GmbH, Schwarzschildstraße 2, Berlin 12489, Germany

**Keywords:** ALD, CVD, Plasma polymer

## Abstract

A widely used application of the atomic layer deposition (ALD) and chemical vapour deposition (CVD) methods is the preparation of permeation barrier layers against water vapour. Especially in the field of organic electronics, these films are highly demanded as such devices are very sensitive to moisture and oxygen. In this work, multilayers of aluminium oxide (AlO _*x*_) and plasma polymer (PP) were coated on polyethylene naphthalate substrates by plasma-enhanced ALD and plasma-enhanced CVD at 80â„ƒ in the same reactor, respectively. As precursor, trimethylaluminium was used together with oxygen radicals in order to prepare AlO _*x*_, and benzene served as precursor to deposit the PP. This hybrid structure allows the decoupling of defects between the single AlO _*x*_ layers and extends the permeation path for water molecules towards the entire barrier film. Furthermore, the combination of two plasma techniques in a single reactor system enables short process times without vacuum breaks. Single aluminium oxide films by plasma-enhanced ALD were compared to thermally grown layers and showed a significantly better barrier performance. The water vapour transmission rate (WVTR) was determined by means of electrical calcium tests. For a multilayer with 3.5 dyads of 25-nm AlO _*x*_ and 125-nm PP, a WVTR of 1.2 × 10 ^−3^ g*m*^−2^*d*^−1^ at 60â„ƒ and 90% relative humidity could be observed.

## Background

Organic optoelectronic devices provide interesting features as they can be applied on inexpensive and flexible large-area substrates [1-3]. However, these devices tend to degrade if they are exposed to atmospheric oxygen and moisture [4-7]. Aluminium (Al), a commonly used electrode material for organic light-emitting diodes (OLEDs) and organic solar cells, is known to have suitable permeation barrier properties [8]. But unfortunately, it is hard to deposit the electrode without any local defects which are mainly caused by particles formed during the deposition process. The defects serve as gas diffusion paths into the device. Oxygen and water molecules can move through these imperfections and then diffuse along the interface between electrode and organic material as well as into the last named. At the interface, oxygen reacts with Al in the following way:

(1) $$4\text{Al}+3O_{2}\to 2{\text{Al}}_{2}O_{3}.$$

The oxide locally insulates the subjacent organic layers, and due to their very low shunt conductivity, they become electrically inactive. The reaction with water is even more critical [7]:

(2) $$2H_{2}O+2e^{-}\to H_{2}+2OH^{-}.$$

The occurrence of hydrogen bubbles around the defects leads to a delamination of the electrode. The emerging hollow space furthermore accelerates the diffusion of water vapour. To suppress the described deteriorations, a reliable encapsulation of organic devices is absolutely necessary for long-term applications. In particular, OLEDs require very low permeation rates as the defects become visible as dark spots at a certain size. In the past, a water vapour transmission rate (WVTR) in the range of 10 ^−6^ g*m*^−2^*d*^−1^ was postulated as an upper limit [9]. This shall ensure a device lifetime of at least 10,000 operating hours. For organic solar cells, the degradation mechanisms are quite similar. However, since the local defects stay invisible as the device does not emit light, the barrier requirements can differ from that of OLEDs. In some cases, a WVTR of 10 ^−3^ g*m*^−2^*d*^−1^ may already be sufficient [10].

A common way to encapsulate a device is to use a glass or metal lid, mounted with an ultraviolet-cured epoxy. Additionally, a desiccant can be used to absorb moisture which can diffuse only through the glue. However, this also implicates some drawbacks. The employment of a glass lid on a flexible OLED, for instance, is not reasonable due to the inelasticity of glass. In addition, the heat accumulation, arising from the poor thermal conductivity of glass, causes a reduced lifetime of the device [11]. If utilised on a top-emitting OLED, which emits its light through the lid, the appearing waveguide losses reduce the external quantum efficiency without special treatments [12]. The prementioned issues are serious reasons to replace this encapsulation approach by thin film barrier layers.

For this purpose, atomic layer deposition (ALD) turned out to be an appropriate tool for fabricating nearly defect-free thin films with excellent gas barrier properties [13]. First and foremost, aluminium oxide (AlO _*x*_) layers have emerged as a suitable thin film encapsulation [14,15]. To deposit ALD films, an alternating inlet of precursors into the reactor chamber takes place. Between the single injections, the reactor is purged with an inert gas to remove redundant precursor molecules and by-products. This inhibits vapour phase reactions and allows a very homogeneous and self-limiting film growth within one reaction cycle [16]. Additionally, plasma-enhanced atomic layer deposition (PEALD) reduces the process time at temperatures below 100â„ƒ since there is no need to remove residual water molecules. Furthermore, for AlO _*x*_, a higher growth per cycle (GPC) can be achieved compared to the thermal ALD (TALD) process. A benefit of hybrid multilayers (ML) is that the separation into several oxide layers leads to a decoupling of morphological defects, e. g. caused by particles, which prolongs the permeation path trough the barrier [8]. A more detailed introduction into moisture barrier layers is given elsewhere [17].

A popular method to measure the WVTR of permeation barriers is the electrical calcium test [18-20]. Calcium (Ca) heavily hydroxylates at contact with water. At temperatures below 70â„ƒ, the dominating reaction is

(3) $$\text{Ca}+2H_{2}O\to \mathrm{Ca}{\left(\text{OH}\right)}_{2}+H_{2}.$$

An oxidation caused by molecular oxygen can be neglected [21-23]. Whereas pure calcium has a good electrical conductivity, Ca(*OH*)_2_ is an insulator. If a current is applied to a thin calcium film, its corrosion can easily be detected as a change of the resistance which allows an immediate calculation of the WVTR.

Since the deposition of hybrid multilayers by TALD/plasma-enhanced chemical vapour deposition (PECVD) has already been shown [24], in this paper, the preparation of MLs by PEALD/PECVD, carried out in one reactor, will be demonstrated. The WVTRs of moisture barrier layers were measured with electrical Ca tests. A correlation of the barrier performance of aluminium oxide layers and their impurity content will also be discussed.

## Methods

### Sample preparation

In order to determine the WVTR, the thin film of interest was coated on a 200- *μ*m-thick polyethylene naphthalate substrate (Teonex Q65, DuPont Teijin Films, Luxembourg) with a size of 25 × 25 m*m*^2^. The polymer foils were cleaned before with acetone, isopropanol and ultrasonic treatments. Prior to deposition, the substrates were stored in the reactor for 72 h at 120â„ƒ to remove residual water in the polymer.

### Layer deposition

The AlO _*x*_ and the plasma polymer (PP) films were deposited in a newly developed plasma system from SENTECH Instruments (patent pending), placed in an ISO class 6 clean room environment. The system was developed and designed for both inductively coupled plasma-enhanced chemical vapour deposition (ICPECVD) and ALD in the same reactor using flexible system architecture. The used plasma source is an inductively coupled planar triple spiral antenna (ICP PTSA 200). A high radio-frequency current flows from the centre through the three arms to the periphery and induces the electric field for generating the high-density plasma [25]. Besides the deposition of AlO _*x*_ and PP layers, the system enables ICPECVD of high-quality oxides and nitride films at low temperatures (80â„ƒ to 130â„ƒ) on different substrate types and sizes. This combination allows the deposition of layer stacks from ALD with low growth rate and ICPECVD with high growth rate in the same chamber.

Reactor walls as well as the substrates were heated to 80â„ƒ, and nitrogen (40 sccm) was applied as carrier and purge gas for trimethylaluminium (TMA) and benzene. The process pressure for the coating of AlO _*x*_ and PP was 12 and 3 Pa, respectively. During the AlO _*x*_ process, the oxygen flow was set to 150 sccm. One AlO _*x*_ deposition cycle included the following steps: 10-s plasma pulse (400 W), 1-s purge time, 0.08-s TMA pulse time and 20-s purge time. The recipe for the PP worked as follows: 0.02-s benzene pulse time, instantly followed by 4-s plasma pulse (200 W) and 6-s purge time. In order to improve the smoothness of PP films, a mass flow of 40 sccm argon was applied. PP-benzene as spacer layer was chosen simply because it allows a rapid film growth. Because of the high vapour pressure of benzene, neither active bubbling nor heating is necessary. One ML dyad is composed of 25-nm PEALD aluminium oxide, which is deposited at first, and 125-nm PECVD PP. x.5 dyads means that the ML is covered with 25-nm PEALD AlO _*x*_ on top. The precursor containers for TMA and benzene were kept at room temperature.

### Calcium test

**Figure 1:**
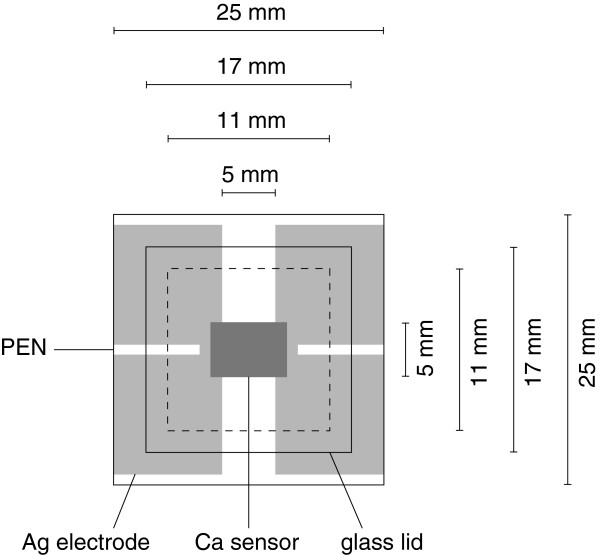
Scheme top view of the electrical calcium test sensor.

After being coated with a multilayer, the polyethylene naphthalate (PEN) substrates were transported into an ultra-high vacuum cluster system with a base pressure of 5 × 10 ^−5^ Pa and stored over night to degas. Afterwards, silver electrodes (100 nm) were prepared by thermal evaporation at a deposition rate of 1.5 Å/s. Ca films with an initial thickness of 100 nm were thermally evaporated at 0.5 Å/s. The active area of the sensor between the electrodes is 5 × 5 m*m*^2^. The aperture of the sensor is given by the glass lid and its cavity (11 × 11 m*m*^2^), which is mounted with an ultraviolet-cured epoxy resin (DELO-KATIOBOND LP686, DELO Industrial Adhesives, Windach, Germany). A schematic of the test setup is shown in Figure 1. The measurement signal was detected by the Kelvin sensing method to eliminate the influence of wire and contact resistances. Therefore, a current of 0.5 mA was applied in order to record one reading per minute with a digital source meter (Keithley 2400, Keithley Instruments Inc., Cleveland, OH, USA) and a four-wire scanning card (Keithley 7067). The WVTR was calculated by means of the formula

(4) $$\text{WVTR}=-2k\frac{l^{2}}{A_{\text{aperture}}}\frac{M_{H_{2}O}}{M_{\mathrm{Ca}}}\delta \rho \frac{d\frac{1}{R}}{\text{dt}}.$$

The factor 2 takes into account that water is the only species in our setup Ca reacts with [18]. *k* includes the fact that the Ca sensor overlaps the electrodes a little. These areas absorb humidity, but their corrosion does not affect the measured voltage. *A* is the area of the aperture, given by the glass lid, and *l* is the length as well as the width of the Ca sensor. *M* is the molar mass of calcium and water, and *δ* and *ρ* are the density and conductivity of calcium, respectively. *R* is the resistance of the sensor. The additional impact of the PEN and Ag electrodes on the total WVTR is insignificant and therefore neglected in the calculation. The resulting steady-state WVTRs were composed of the average of four samples. To accelerate the measurement, the tests were performed in a climate cabinet (Binder KBF 115, BINDER GmbH, Tuttlingen, Germany) at 60â„ƒand 90% relative humidity (RH). These conditions naturally lead to higher permeation rates than measurements at room temperature.

### Analytics

The carbon (C) content of different AlO _*x*_ layers was detected with energy-dispersive X-ray spectroscopy (JEOL JSM 6400, JEOL Ltd., Tokyo, Japan) at a beam energy of 7 kV. In order to control the growth per cycle, the total thickness as well as the refractive index of the films, deposited on silicon substrates with native oxide, was measured with spectroscopic ellipsometry (GES5, Semilab Semiconductor Physics Laboratory Co. Ltd., Budapest, Hungary) and then divided by the number of process cycles. The surface roughness was determined by atomic force microscopy (AFM) with a DME DualScope DS 45-40 (Danish Micro Engineering A/S DME, Herlev, Denmark).

## Results and discussion

**Figure 2:**
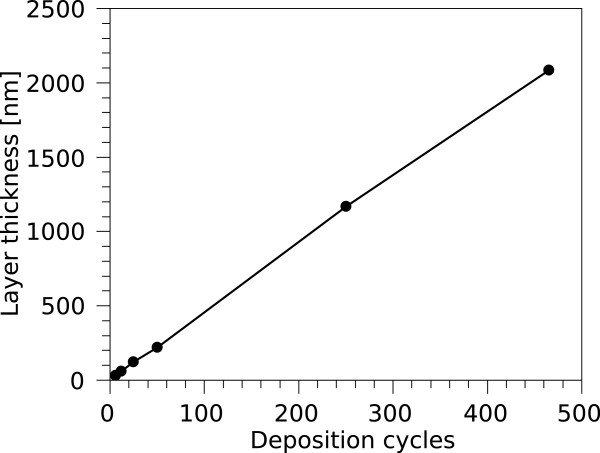
Layer thickness over deposition cycles of the PECVD plasma polymer growth.

**Figure 3:**
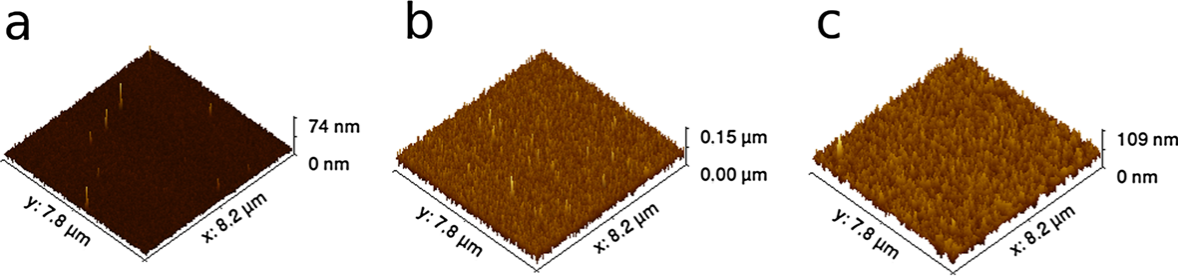
**AFM images. (a)** 25-nm PEALD aluminium oxide and **(b)** 125-nm PECVD PP sublayers and **(c)** a AlO _*x*_/PP multilayer with 2.5 dyads. All samples were coated on silicon substrates with native oxide.

**Figure 4:**
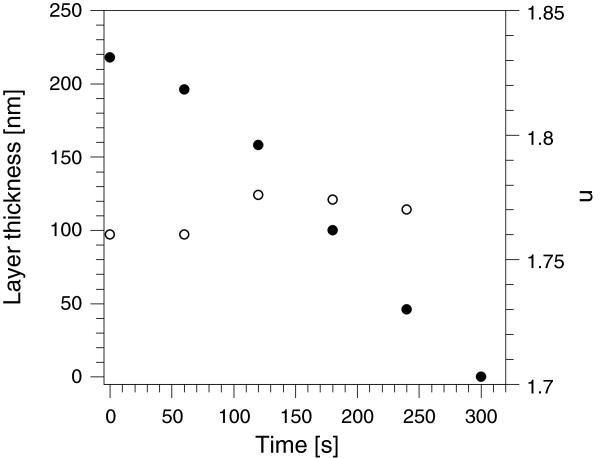
**Layer thickness and refractive index.** Decreasing layer thickness (filled circles) and refractive index at 633 nm (empty circles) of a PP sample in oxygen plasma as a function of time.

The PECVD process for fabricating PP films was carried out in a non-continuous mode, similar to ALD cycles. The growth per cycle (GPC) is 4.5 nm/cycle which is equivalent to 27 nm/min and very constant up to a layer thickness of more than 2 µm, as shown in Figure 2. The chemical structure of PP-benzene by PECVD can be found elsewhere [26]. Aluminium oxide films were grown with a GPC of 0.18 nm/cycle. The root mean square (RMS) of an AlO _*x*_ sublayer was derived from AFM images, as shown in Figure 3a. With a RMS value of 0.3 nm, the oxide layer turned out to be very smooth. The surface of PP sublayers had a RMS of 0.9 nm (Figure 3b). Figure 3c displays the surface of a multilayer with 2.5 dyads with a measured RMS of 1.5 nm. The investigated multilayers were built up of 1.5, 2.5 and 3.5 dyads. For a ML with 3.5 dyads, the calculated thickness is 475 nm, but instead, only 399 nm was measured. This leads to the assumption that an etching of the PP through the oxygen plasma took place. According to Figure 4, which shows the removing of a PP sample with an initial thickness of 220 nm on silicon in an O _2_ plasma (with the same parameters as for the PEALD process), the etch rate is roughly 1 nm/s. This process must appear during the very first PEALD cycles and stops when AlO _*x*_ forms a continuous film. Hence, the sublayer thickness of PP is rather 100 nm than 125 nm. The refractive index merely changed slightly during O _2_ plasma treatment and a significant densification of the polymer is therefore rather unlikely (see Figure 4). A change of the surface roughness after 60 s in O _2_ plasma did not occur. When coating 50-nm TALD AlO _*x*_ on top of a PP layer, a decreasing of the PP thickness could not be observed.

Table 1 provides an overview of the moisture barrier performance of different hybrid multilayers. Moreover, the MLs were compared with a glass lid encapsulation, where the coated PEN was substituted by a glass substrate, and single aluminium oxide layers. The latter was plasma enhanced and thermally grown, respectively. The TALD AlO _*x*_ sample was fabricated with a Savannah 200 ALD tool (Cambridge Nanotech, Cambridge, MA, USA) at 80â„ƒ with a GPC of 0.12 nm/cycle. PEALD AlO _*x*_, grown at 400 W and 10-s pulse time, shows with 4.4 × 10 ^−3^ g*m*^−2^*d*^−1^, a significantly better barrier performance than samples deposited at 100 W and 1-s pulse time and TALD AlO _*x*_ films with the same layer thickness. A possible reason for this phenomenon will be discussed later. A ML with 1.5 dyads has the same overall oxide thickness as a single aluminium oxide film. However, its WVTR of 3.6 × 10 ^−3^ g*m*^−2^*d*^−1^ is slightly lower. Although the difference is quite small, this might be a result of the splitting of one AlO _*x*_ film into two layers in order to separate local defect paths. Continuing the stacking of dyads led to a further improvement of the WVTR. With 3.5 dyads, a transmission rate of 1.2 × 10 ^−3^ g*m*^−2^*d*^−1^ could be realised. This value is only by a factor of 2 higher as the one of a glass lid encapsulation. The lag time, which is the time elapsing until the phase of steady-state arises, increased from approximately 55 h at 1.5 dyads to approximately 97 h at 3.5 dyads due to the extended pathways for water through the ML. At 3.5 dyads, the overall oxide thickness is twice as large as at 1.5 dyads. However, the WVTR is lower by a factor of 3. In contrast, doubling the layer thickness of TALD AlO _*x*_ to 100 nm merely enhanced the permeation rate of about 20% (6.4 × 10 ^−3^ g*m*^−2^*d*^−1^), whereas reducing the thickness to 25 nm increases the WVTR by more than 1 order of magnitude (Table 2). This large rise may be attributed by the fact that not all particles and defects on the PEN surface are fully covered on the one hand and still remaining water in the substrate, which influences the first nanometre of layer growth on the other hand. With continuing film growth, only defects with sizes >100 nm persist uncovered and dominate the permeation process, as the WVTR merely changes from 50 to 100 nm.

**Table 1:** **WVTRs with mean deviation of several AlO **_***x***_**/PP multilayers and single AlO **_***x***_** films, measured at 60â„ƒ and 90% RH**

**Barrier**	**WVTR [g*m***^**−2**^***d***^**−1**^**]**
Glass lid	(6 ± 2) × 10 ^−4^
3.5 dyads	(1.2 ± 0.7) × 10 ^−3^
2.5 dyads	(2 ± 0.9) × 10 ^−3^
1.5 dyads	(3.6 ± 1.3) × 10 ^−3^
50-nm PEALD aluminium oxide (400 W, 10 s)	(4.4 ± 0.8) × 10 ^−3^
50-nm PEALD aluminium oxide (100 W, 1 s)	(8.5 ± 2.4) × 10 ^−3^
50-nm TALD aluminium oxide	(7.7 ±2.3) × 10 ^−3^

**Table 2:** WVTRs with mean deviation of TALD aluminium oxide films with layer thicknesses from 25 to 100 nm, measured at 60â„ƒ and 90% RH

**Thickness [nm]**	**WVTR [g*m***^**−2**^***d***^**−1**^**]**
25	(8.5 ± 2.2) × 10 ^−2^
50	(7.7 ± 2.3) × 10 ^−3^
100	(6.4 ±1.2) × 10 ^−3^

In order to investigate the correlation between process conditions and barrier performance, the carbon content of different aluminium oxide films, given in Table 3, was detected by energy-dispersive X-ray spectroscopy (EDX). All samples had a layer thickness of 150 nm to achieve sufficient measuring signals. It may be worthy to note that the hydrogen atoms cannot be traced by EDX, and that is why the unit weight percent (wt.%) is used instead of atomic percent (at.%). To exclude a contamination of the analytical chamber, a clean silicon wafer was also investigated. Its carbon content was determined to be 0 wt.%. The data expose a relation between the process conditions and the carbon content. Longer plasma pulse times lead to significantly lower impurities. At 400 W, an elongation of the pulse time from 1 to 10 s clearly reduces the residual carbon from 6 to 3.1 wt.%. But the plasma power also has an impact on the composition of the AlO _*x*_ films. The carbon itself probably originates from hydrocarbons due to incomplete surface reactions [27,28]. The thermally grown AlO _*x*_ had a C content of 4.6 wt.%, which is more than the best plasma-assisted grown film included (3.1 wt.% at 400 W and 10-s pulse time). A thermally grown aluminium oxide film at 200â„ƒ exhibited a C content of only 2.2 wt.% which may also be attributed to a lower content of hydrocarbons in the film. It is known from previous researches that in low-temperature and low-power PALD aluminium oxide films, respectively, hydroxy groups are also contained in a significant amount, resulting in a lower film density [29]. Albeit the change of the refractive indices, also given in Table 3, is quite small, it can serve as an indicator as well that increasing the amount of oxygen radicals can lead to denser films. It is believed that both types of impurity allow water molecules not only to walk through pinholes or cracks but also to diffuse through the AlO _*x*_ itself.

**Table 3:** Carbon content and refractive index at 633 nm of aluminium oxide films at different process conditions, deposited at 80â„ƒ

**Plasma power [W]**	**Plasma pulse time [s]**	**C [wt.%]**	***n***
400	10	3.1	1.62
400	1	6	1.60
100	10	4.6	1.61
100	1	7	1.60
Thermally grown		4.6	1.60

## Conclusions

A combination of a PEALD and PECVD process in one reactor chamber was demonstrated in order to accelerate the fabrication of thin moisture barrier layers with a high film quality. For hybrid multilayers of 3.5 dyads, a steady-state WVTR of 1.2 × 10 ^−3^ g*m*^−2^*d*^−1^ at 60â„ƒ and 90% RH could be achieved, which is nearby the value of a glass lid encapsulation. At optimised process conditions, a single PEALD aluminium oxide layer revealed a better barrier performance than a thermally grown one, which is probably associated with a lower incorporation of hydrocarbons and hydroxyl groups, respectively.

## Competing interests

The authors declare that they have no competing interests.

## Authors’ contributions

TB drafted the manuscript and carried out the experiments as well as the analyses, and participated in the design of the study. HG wrote parts of the manuscript and supervised the study. MS and RR developed the utilised deposition system. HHJ participated in the design of the study and supervised it. WK is in charge of the project and supervised it. All authors read and approved the final manuscript.
